# Differing Outcome of Experimental Autoimmune Encephalitis in Macrophage/Neutrophil- and T Cell-Specific gp130-Deficient Mice

**DOI:** 10.3389/fimmu.2018.00836

**Published:** 2018-05-02

**Authors:** Kristian Holz, Marco Prinz, Stefanie M. Brendecke, Alexandra Hölscher, Fengyuan Deng, Hans-Willi Mitrücker, Stefan Rose-John, Christoph Hölscher

**Affiliations:** ^1^Division of Infection Immunology, Research Center Borstel, Borstel, Germany; ^2^Institute of Neuropathology, Medical Faculty University of Freiburg, Freiburg, Germany; ^3^BIOSS Center for Biological Signaling Studies, University of Freiburg, Freiburg, Germany; ^4^Institute of Immunology, University Medical Center Hamburg-Eppendorf, Hamburg, Germany; ^5^Department of Biochemistry, Christian-Albrechts-University, Kiel, Germany; ^6^Cluster of Excellence Inflammation-at-Interfaces, Borstel-Kiel-Lübeck-Plön, Germany; ^7^Priority Area Infection, Research Center Borstel, Borstel, Germany

**Keywords:** experimental autoimmune encephalomyelitis/multiple sclerosis, gp130 cytokine, interleukin-6, interleukin-27, inflammation mediators

## Abstract

gp130 cytokines are differentially involved in regulating the T helper (H) 17-driven pathogenesis of experimental autoimmune encephalomyelitis (EAE), the animal model of human multiple sclerosis. Interleukin (IL)-6 directly promotes the development of TH17 cells through the gp130/IL-6R complex. By contrast, IL-27 has been shown to suppress a TH17 immune response by gp130/IL-27R-alpha (α) receptor ligation. The IL-27-dependent regulation of a TH17 development could be mediated on the level of CD4 T cells. However, because IL-27 also suppresses the secretion of the TH17-driving cytokines IL-6 and IL-12/23p40 in accessory cells, TH17 immune responses may also be controlled by IL-27 on the level of macrophages and/or neutrophils. To analyze these opposing effects of gp130 engagement on the pathogenesis of EAE, we immunized CD4^+^ T cell-specific gp130-deficient (CD4cre^pos^gp130^loxP/loxP^) and macrophage/neutrophil-specific gp130-deficient (LysMcre^pos^gp130^loxP/loxP^) mice with the myelin-oligodendrocyte-glycoprotein peptide MOG_35–55_. Whereas inflammatory immune responses, TH17 differentiation, and pathology in CD4cre^pos^gp130^loxP/loxP^ mice were mitigated, disease progression was eventually enhanced in LysMcre^pos^gp130^loxP/loxP^ mice. Exacerbated disease in MOG_35–55_-immunized LysMcre^pos^gp130^loxP/loxP^ mice was associated with an elevated development of TH17 cells and increased infiltration of the central nervous system with leukocytes indicating a suppressive role of macrophage/neutrophil-gp130. To further prove IL-6 to be responsible for the control of inflammation during EAE through gp130 on macrophages/neutrophils, we immunized LysMcre^pos^IL-6R^loxP/loxP^ mice. In contrast to LysMcre^pos^gp130^loxP/loxP^ mice, neuropathology in MOG_35–55_-immunized macrophage/neutrophil-specific IL-6R-deficient mice was not enhanced indicating that the alleviation of EAE through macrophage/neutrophil-gp130 is mediated independently of IL-6. Together, this different pathology in macrophage/neutrophil- and CD4 T cell-specific gp130-deficient mice suggests that gp130 cytokines modulate TH17 inflammation differentially by targeting distinct cell types.

## Introduction

Multiple sclerosis (MS) is one of the most common neurological diseases worldwide. It is well documented that MS represents an inflammatory demyelinating disease of the central nervous system (CNS) which is mediated by pathogenic CD4^+^ T helper (H) cells (1). Using the animal model for MS known as experimental autoimmune encephalomyelitis (EAE), recent studies could show that autoreactive myelin-specific TH17 cells are responsible for the induction and maintenance of neuroinflammation whereas regulatory T cells (T_reg_) represent the most relevant cell line with respect to EAE resistance (2). Originally named for their ability to produce interleukin (IL)-17A and IL-17F, TH17 cells are crucial for the amplification of neuroinflammation by activating epithelial cells and attracting other immune cells because of their ability to express additional pro-inflammatory mediators such as tumor necrosis factor (TNF), granulocyte colony stimulating factor, granulocyte macrophage colony stimulating factor, IL-21, or IL-22 (3, 4). Since the discovery of TH17 cells, numerous studies have focused on attenuating the impact of pathogenic TH17 cells as a therapeutic strategy (5–7).

In the EAE model, the initial differentiation of autoreactive myelin-specific TH17 cells is induced in lymphoid organs *via* immunization with an emulsion of the complete Freund’s adjuvant (CFA) and the myelin-oligodendrocyte-glycoprotein peptide (MOG)_35–55_. Comparative analyses of gene-deficient mice showed that especially the pro-inflammatory cytokine IL-6 together with TGFβ is considered the most important pro-inflammatory mediator for the development of TH17 cells (8). This has convincingly been shown by using IL-6-deficient (^−/−^) mice, which are completely resistant to EAE (9–11). By contrast, in the absence of IL-6 secretion, the sole presence of TGFβ leads to the development of T_reg_ (12–16). Therefore, IL-6 that uses the gp130/IL-6R receptor complex for signaling constitutes a key role because it first suppresses the development of T_reg_ and on the other hand directly induces the development of pathogenic TH17 cells (12, 17).

In addition to the gp130 cytokine IL-6, the heterodimeric cytokine IL-27 also uses the receptor subunit gp130 for signaling (18). However, binding to the gp130/IL-27R-alpha (α) receptor complex IL-27 mediates inhibitory effects on the development of pathogenic TH17 cells and therefore acts contrary to the pro-inflammatory cytokine IL-6 (19–21). In addition, antagonizing gp130 signaling by overexpression of IL-27p28 *in vivo* also ameliorated EAE pathology and reduced tissue infiltration due to decreased lineage stability of effector T cells (22, 23). Thus, IL-27 plays a crucial role in protection against EAE development. In fact, the induction of EAE in IL-27Rα^−/−^ mice led to a significant increase in neuropathology which was accompanied by an enhanced expression of pro-inflammatory cytokines (24, 25). Hence, in the EAE model the gp130 cytokines IL-6 and IL-27 exert diametrically opposed effects on the development of TH17 cells.

Whereas gp130 is ubiquitously expressed, the cell type-specific effects of IL-6 and IL-27 signaling relies on the expression of the private cytokine-specific receptor subunits IL-6R and IL-27Rα, respectively. In addition to CD4^+^ T cells, activated macrophages and neutrophils are also capable of expressing IL-6R and IL-27Rα together with gp130 (26–32). However, not much is known about the effect of gp130 cytokines on these cells. Macrophage/neutrophil-gp130 has been shown to modulate the dynamics of innate immune cell recruitment and activation in the acute stages of intestinal inflammation (30). On the other hand, it has been repeatedly documented that IL-6 as well as IL-27 suppress inflammatory immune responses of macrophages (26–29, 31, 32). In addition, IL-27 also modulates neutrophil development and function (33–35). Thus, IL-6 and IL-27 exhibit essential regulatory functions and consequently indirectly modulate inflammatory immune responses. Therefore, gp130 cytokines also may indirectly regulate adaptive immune responses during the course of EAE by modulating the secretion of inflammatory mediators by macrophages.

To elucidate the differential function of T cell-gp130 and macrophage/neutrophil-gp130 on the development of EAE, conditional gp130^loxP/loxP^ mice were crossed with T cell-specific CD4cre^pos^ and macrophages/neutrophil-specific lysozyme (Lys) Mcre^pos^ deleter mice. After immunization with MOG_35–55_/CFA, the development of EAE in CD4cre^pos^gp130^loxP/loxP^ mice and LysMcre^pos^gp130^loxP/loxP^ mice was analyzed in comparison with the respective cre-negative littermates. To further analyze macrophage/neutrophil-specific effects on neuropathology mediated by IL-6, we also included immunized LysMcre^pos^IL-6R^loxP/loxP^ mice.

## Results

### MOG_35–55_-Immunized CD4cre^pos^gp130^loxP/loxP^ Mice Are Resistant to EAE Induction

**Figure 1:**
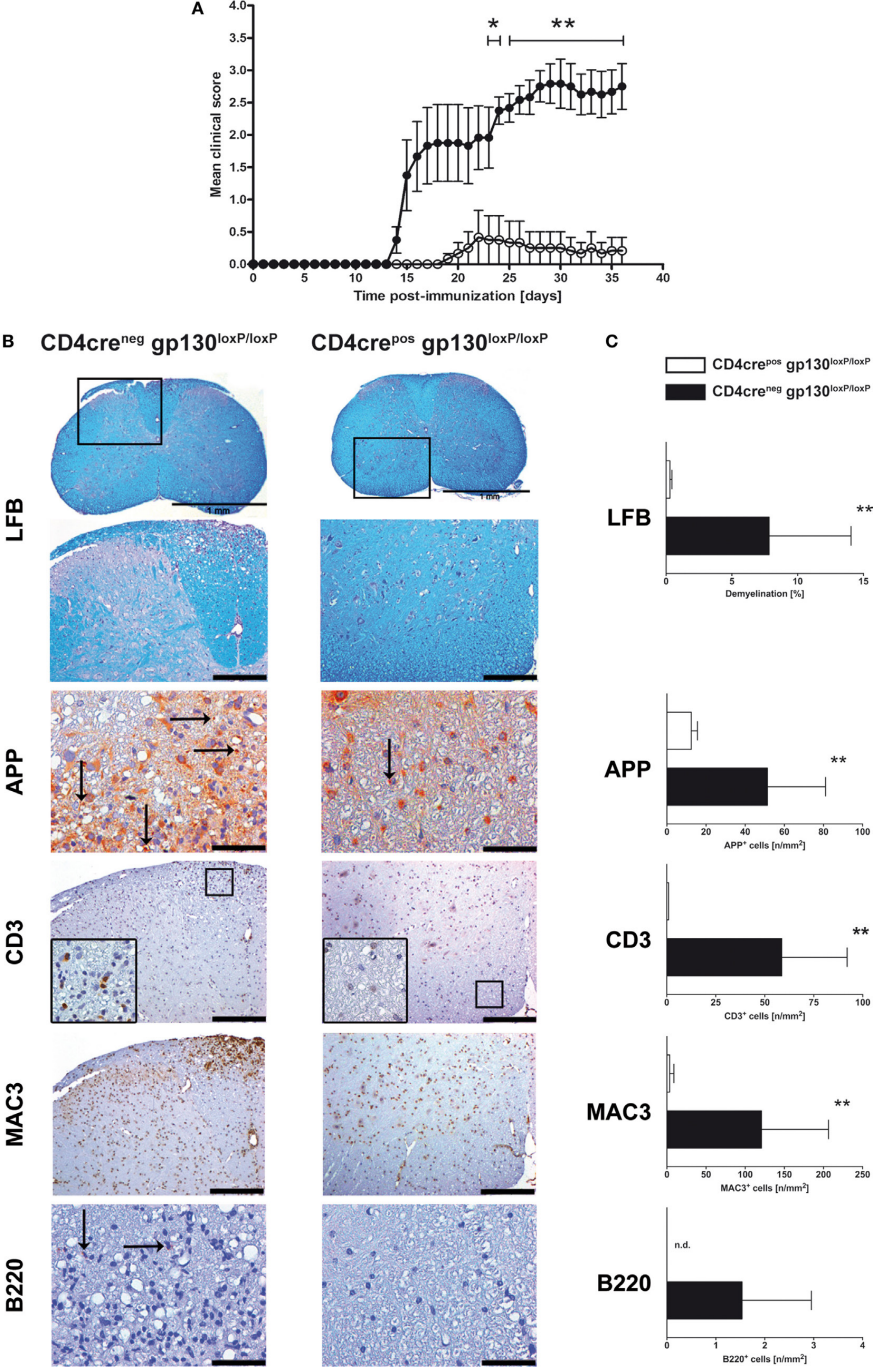
Disease progression and neuropathology in immunized CD4cre^pos^gp130^loxP/loxP^ mice and CD4cre^neg^gp130^loxP/loxP^ mice. Experimental autoimmune encephalomyelitis (EAE) was induced by active immunization of CD4cre^pos^gp130^loxP/loxP^ mice (white circles and bars) and CD4cre^neg^gp130^loxP/loxP^ littermates (black circles and bars). **(A)** Clinical disease of EAE. Data represent means and SEs of six mice per group. One experiment representative out of three performed is shown. **(B,C)** 21 days after immunization, histopathological and immunohistochemical analysis of spinal cord sections was performed. Panel **(B)** shows regions of demyelination [luxol fast blue (LFB)], distribution of amyloid precursor protein (APP) deposits representing axonal damage, and presence of CD3^+^, MAC3^+^, and B220^+^ cells. If not indicated, scale bars represent 50 µm (APP) and 200 µm (LFB, CD3, MAC3, and B220). Panel **(C)** shows extent of demyelination, quantity of APP deposits and infiltration of CD3^+^ T cells, MAC3^+^ macrophages, and B220^+^ B cells. Data represent means and SEs of five mice per group. Statistical analysis was performed using Mann–Whitney test. Asterisks indicate statistical significance (*p ≤ 0.05; **p ≤ 0.01). Abbreviation: n.d., not detectable.

gp130 cytokines like IL-6 and IL-27 induce different mechanisms in various cell types. Whereas IL-6 promotes the differentiation of CD4^+^ T cells to TH17 cells, IL-27 suppresses TH17 development of CD4^+^ T cells. Accordingly, both cytokines differentially modulate the development of CD4^+^ T cells to pathogenic TH17 cells during the course of EAE. To elucidate the function of gp130-dependent cytokines on activated T cells, conditional gp130^loxP/loxP^ mice were crossed with T cell-specific CD4cre^pos^ deleter mice. Examination of the efficient deletion of gp130 in CD4^+^ T cells was performed by flow cytometry of single cell suspension of spleens isolated from CD4cre^neg^ and CD4cre^pos^gp130^loxP/loxP^ mice. Whereas gp130 was found to be readily expressed on CD4^+^ T cells of CD4cre^neg^ mice, it was nearly absent on CD4^+^ T cells of CD4cre^pos^gp130^loxP/loxP^ mice (Figure S1 in Supplementary Material). To analyze the impact of gp130 signaling on T cells for the development of EAE, CD4cre^pos^gp130^loxP/loxP^ mice and CD4cre^neg^gp130^loxP/loxP^ littermates were immunized with MOG_35–55_ emulsified in CFA and monitored for EAE symptoms. Upon immunization, all CD4cre^neg^gp130^loxP/loxP^ mice developed neurological symptoms such as tail weakness and paralysis, starting 2 weeks after EAE induction (mean maximal score: 3.04 ± 0.64) (Figure 1A). By contrast, CD4cre^pos^gp130^loxP/loxP^ littermates showed significantly reduced clinical signs and were almost resistant to the induction of EAE (mean maximal score: 0.42 ± 1.02). To assess demyelination and axonal damage within the CNS of diseased mice, histological analyses of spinal cord sections were carried out 21 days after immunization (Figure 1B). The extent of demyelination and the loss of axons were detected by luxol fast blue (LFB) staining and by staining of amyloid precursor protein (APP) deposits. Upon quantification, immunized CD4cre^pos^gp130^loxP/loxP^ mice showed significantly less regions of demyelination as well as fewer APP^+^ deposits compared with cre-negative littermates (Figure 1C). Infiltration of the CNS with leukocytes is key to the pathology of EAE. We therefore performed immunohistochemical staining of CD3^+^ T cells, MAC3^+^ macrophages, and B220^+^ B cells (Figure 1B). Quantification of the accumulation of these cells revealed that the infiltration in CD4cre^pos^gp130^loxP/loxP^ mice was significantly reduced (Figure 1C). Thus, this distinct neuropathology observed in histological analyses of immunized CD4cre^pos^gp130^loxP/loxP^ and CD4cre^neg^gp130^loxP/loxP^ mice reflected the development of clinical EAE symptoms after immunization.

### Immunized CD4cre^pos^gp130^loxP/loxP^ Mice Show Significantly Reduced TH17 and TH1 Development

**Figure 2:**
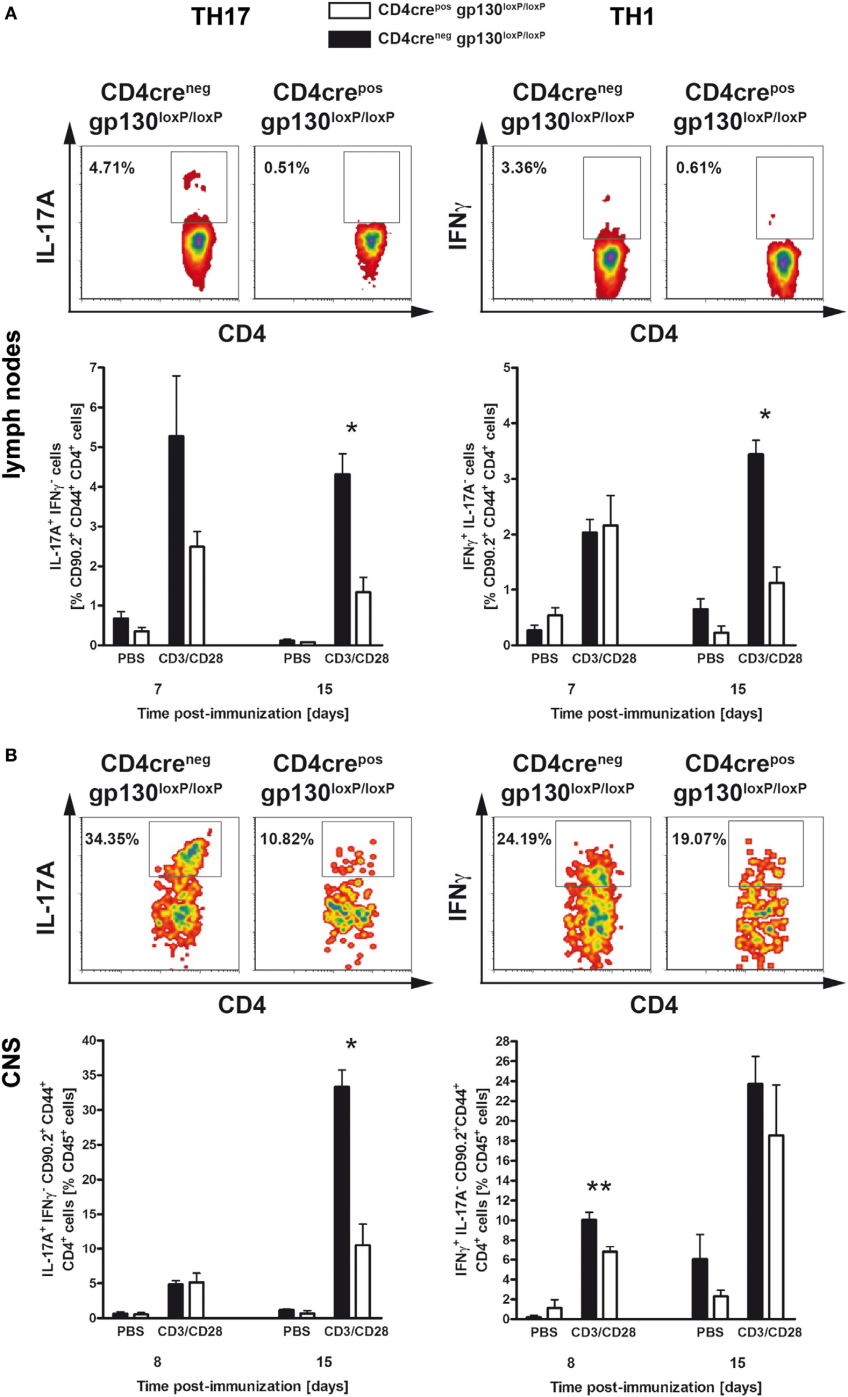
TH17 and TH1 immune responses in immunized CD4cre^pos^gp130^loxP/loxP^ mice and CD4cre^neg^gp130^loxP/loxP^ littermates. CD4cre^pos^gp130^loxP/loxP^ (white bars) mice and CD4cre^neg^gp130^loxP/loxP^ (black bars) littermates were immunized with MOG_35–55_/CFA. During the course of EAE, the frequency of TH17 and TH1 cells was determined by intracellular cytokine staining and subsequent flow cytometry. Panels **(A,B)** show analysis of intracellular interleukin (IL)-17A (left) and IFNγ production (right) by anti-CD3/CD28-stimulated cells. For the analysis of TH17 and TH1 cells, single cell suspensions of **(A)** draining lymph nodes and **(B)** central nervous system (CNS)-infiltrating lymphocytes were performed at different time points (gated on CD90.2^+^CD44^+^CD4^+^ cells). Representative flow cytometry density plots of IL-17A- and IFNγ-producing cells isolated on day 15 are shown. Data represent means and SEs of at least three mice **(A)** and five mice **(B)** per group, respectively. **(A,B)** One experiment representative out of three performed is shown. Statistical analysis was performed using Mann–Whitney test. Asterisks indicate statistical significance (*p ≤ 0.05; **p ≤ 0.01).

To analyze the instructive events that account for EAE-susceptibility of immunized CD4cre^pos^gp130^loxP/loxP^ mice and CD4cre^neg^gp130^loxP/loxP^ littermates in more detail, the frequency of TH1 and TH17 cells was determined at different time points early after immunization (Figures 2A,B). Both, in lymph nodes as well as within the CNS, intracellular staining of IL-17A and IFNγ in activated CD4^+^ T cells showed that the development of inflammatory TH17 and TH1 cells was significantly reduced in immunized CD4cre^pos^gp130^loxP/loxP^ mice (Figures 2A,B). More precisely, after restimulation of single cell suspensions with anti-CD3/CD28, the frequency of TH17 cells was dramatically reduced in lymph nodes and spleen of immunized CD4cre^pos^gp130^loxP/loxP^ mice (Figure 2A left). The frequency of TH1 cells was significantly reduced 15 days after immunization (Figure 2A right). Determining the percentage of TH17 and TH1 cells referring to all CNS-infiltrated CD45^+^ cells revealed that the initial infiltration of TH1 cells (day 8) and TH17 cells (day 15) was significantly reduced in CD4cre^pos^gp130^loxP/loxP^ mice compared with cre-negative littermates (Figure 2B).

Collectively, these data indicate that the resistance observed in immunized CD4cre^pos^gp130^loxP/loxP^ mice resulted from reduced neuropathology due to diminished TH17 and TH1 cell development as well as ameliorated CNS-infiltration already at an early stage of the disease. Accordingly, the deletion of gp130 on T cells prevented the development of inflammatory T cells, meaning that the expression of T cell-gp130 is essential for TH17 and TH1 cell development.

### LysMcre^pos^gp130^loxP/loxP^ but Not LysMcre^pos^IL-6R^loxP/loxP^ Mice Show Significantly Increased Disease Progression After MOG_35–55_-Immunization During the Chronic Stage of the Disease

**Figure 3:**
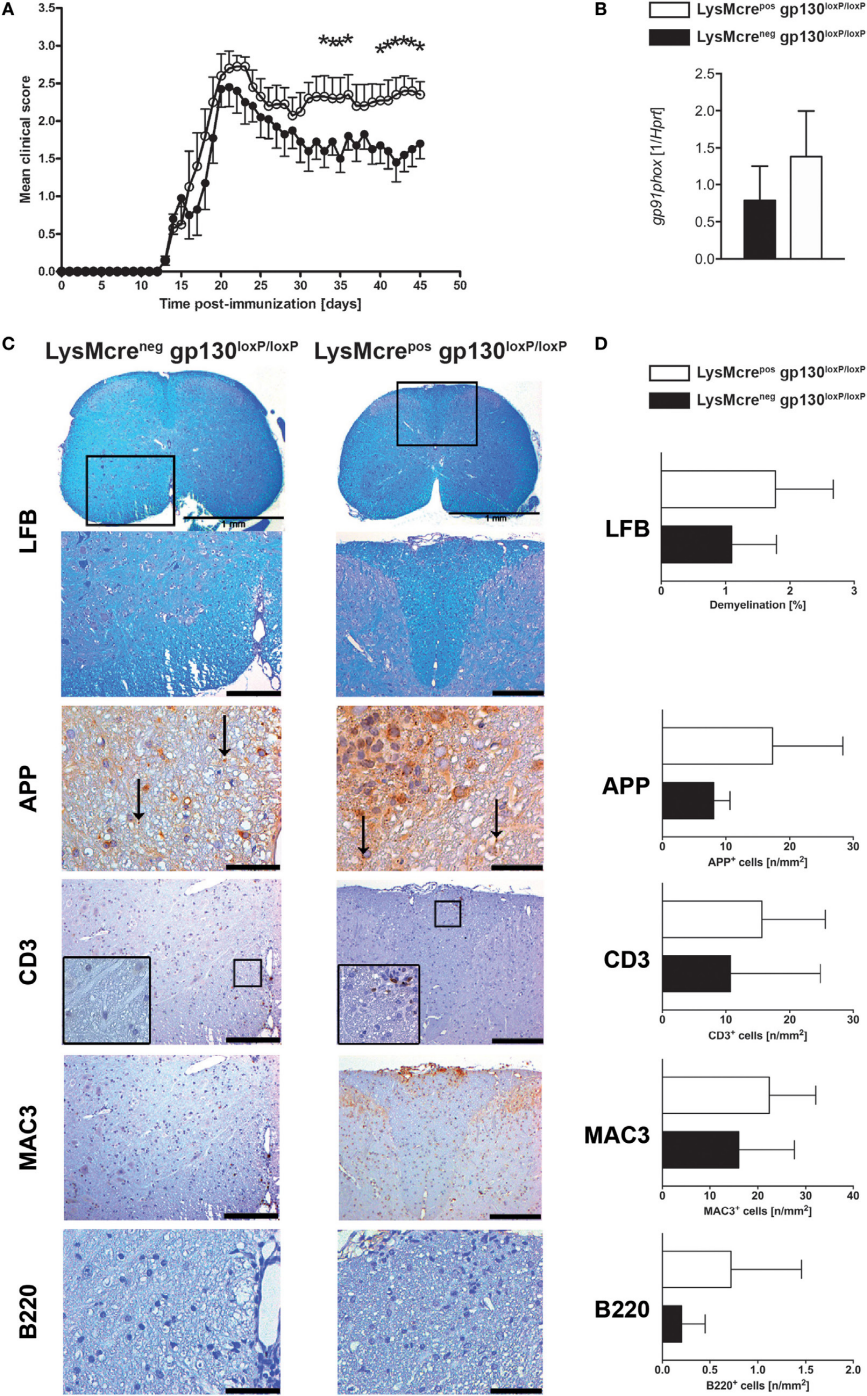
Disease progression and neuropathology in immunized LysMcre^pos^gp130^loxP/loxP^ mice and LysMcre^neg^gp130^loxP/loxP^ mice. LysMcre^pos^gp130^loxP/loxP^ mice (white circles and bars) and LysMcre^neg^gp130^loxP/loxP^ littermates (black circles and bars) were immunized with MOG_35–55_/CFA. **(A)** Clinical course of EAE in LysMcre^pos^gp130^loxP/loxP^ mice compared with LysMcre^neg^gp130^loxP/loxP^ littermates. One experiment representative out of three performed is shown. Data represent means and SEs of 10 mice per group. **(B)** 15 days after immunization, CNS homogenates were assayed for *Gp91phox* gene expression by quantitative real-time PCR. Data are expressed as ratio of *Gp91phox* versus endogenous *Hprt* and expressed as means and SEs of four mice per group. Statistical analysis was performed using Mann–Whitney test. **(C,D)** 21 days after immunization, histopathological and immunohistochemical analysis of spinal cord sections was performed. Panel **(C)** shows regions of demyelination [luxol fast blue (LFB)], distribution of amyloid precursor protein (APP) deposits representing axonal damage and presence of CD3^+^, MAC3^+^, and B220^+^ cells. If not indicated, scale bars represent 50 µm (APP) and 200 µm (LFB, CD3, MAC3, and B220). Panel **(D)** shows extent of demyelination and quantity of APP deposits and of infiltration with CD3^+^ T cells, MAC3^+^ macrophages, and B220^+^ B cells. Data represent means and SEs of five (LysMcre^pos^gp130^loxP/loxP^) and four (LysMcre^neg^gp130^loxP/loxP^) mice per group, respectively. Statistical analysis was performed using Mann–Whitney test. Asterisks indicate statistical significance (*p ≤ 0.05).

gp130 cytokines like IL-6 and IL-27 not only exert distinct functions on activated T cells but they also serve as inhibitors for activated macrophages/neutrophils in a gp130-dependent manner. Therefore, the effect of gp130 cytokines on the development of EAE may also be regulated indirectly by suppressing the TH17-driving cytokine secretion of activated macrophages/neutrophils. To analyze the function of gp130 on macrophages/neutrophils during the course of EAE, conditional gp130^loxP/loxP^ mice were crossed with macrophage/neutrophil-specific LysMcre^pos^ deleter mice. Cre-positive offspring of this breeding was shown to efficiently delete gp130 on macrophages (31). Immunization of LysMcre^pos^gp130^loxP/loxP^ mice and LysMcre^neg^gp130^loxP/loxP^ littermates with MOG_35–55_/CFA demonstrated that the deletion of gp130 on macrophages/neutrophils of LysMcre^pos^gp130^loxP/loxP^ mice resulted in significantly increased susceptibility to EAE (Figure 3A). More precisely, whereas the onset was similar in both mouse strains, the chronic effector phase of the disease was changed, with an enhanced mean maximal score in LysMcre^pos^gp130^loxP/loxP^ mice (3.05 ± 0.37 versus 2.68 ± 0.46 in LysMcre^neg^gp130^loxP/loxP^ littermates). Because oxidative stress appears to be a key mechanism promoting CNS tissue damage in EAE and expression of the gp91phox subunit of the NADPH oxidase complex correlates with the degree of EAE (36), we quantified gene expression of *gp91phox* in the CNS of immunized mice and found that 15 days after immunization the expression of gp91phox was not significantly increased in LysMcre^pos^gp130^loxP/loxP^ mice (Figure 3B). Using LFB staining of spinal cord sections isolated 21 days after immunization, we could not detect a significantly different demyelination of the white matter in LysMcre^pos^gp130^loxP/loxP^ mice compared with cre-negative littermates (Figures 3C,D). In addition, by determining the amount of APP^+^ deposits, we also found no significantly increased axonal damage in these mice at this early time point. Immunohistochemical staining and subsequent quantification of infiltrating CD3^+^ T cells, MAC3^+^ macrophages and B220^+^ B cells did also not reveal an overall significantly increased inflammation in LysMcre^pos^gp130^loxP/loxP^ mice (Figures 3C,D).

**Figure 4:**
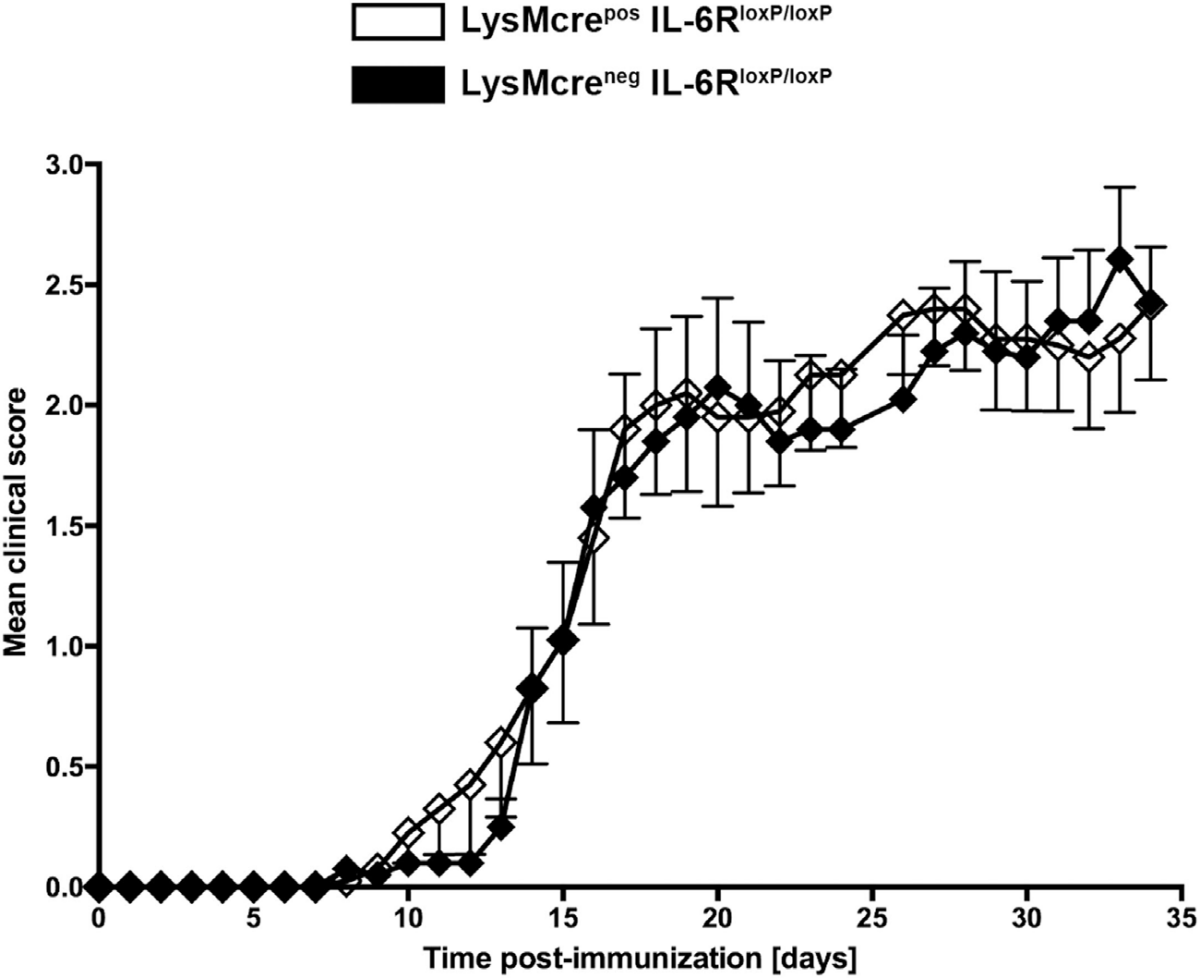
Disease progression in immunized LysMcre^pos^IL-6R^loxP/loxP^ mice and LysMcre^neg^IL-6R^loxP/loxP^ mice. LysMcre^pos^IL-6R^loxP/loxP^ mice (white rhombs) and LysMcre^neg^IL-6R^loxP/loxP^ littermates (black rhombs) were immunized with MOG_35–55_/CFA. Clinical course of EAE in LysMcre^pos^IL-6R^loxP/loxP^ mice compared with LysMcre^neg^IL-6R^loxP/loxP^ littermates. One experiment representative out of two performed is shown. Data represent means and SEs of 10 mice per group. Statistical analysis was performed using Mann-Whitney test.

Because IL-6 and IL-27 both suppress inflammatory immune responses of macrophages (26–29, 31, 32), we were wondering whether either cytokine is responsible for the increased pathology in the absence of macrophage/neutrophil-gp130. To analyze the specific effect of IL-6 on macrophages during EAE, we immunized LysMcre^pos^IL-6R^loxP/loxP^ mice that have been shown to efficiently delete IL-6R on macrophages (37, 38) and LysMcre^neg^IL-6R^loxP/loxP^ littermates with MOG_35–55_/CFA. In contrast to a macrophage/neutrophil-specific gp130 deficiency (see Figure 3A), a deletion of IL-6R on macrophages/neutrophils in LysMcre^pos^IL-6R^loxP/loxP^ mice did not increase the susceptibility to EAE (Figure 4).

In summary, the immunization of LysMcre^pos^gp130^loxP/loxP^ mice and LysMcre^pos^IL-6R^loxP/loxP^ mice with MOG_35–55_/CFA demonstrated that the development of EAE symptoms during the chronic stage of the disease is regulated on the level of activated macrophages/neutrophils.

### Predominant TH17 and TH1 Development in Immunized LysMcre^pos^gp130^loxP/loxP^ Mice

**Figure 5:**
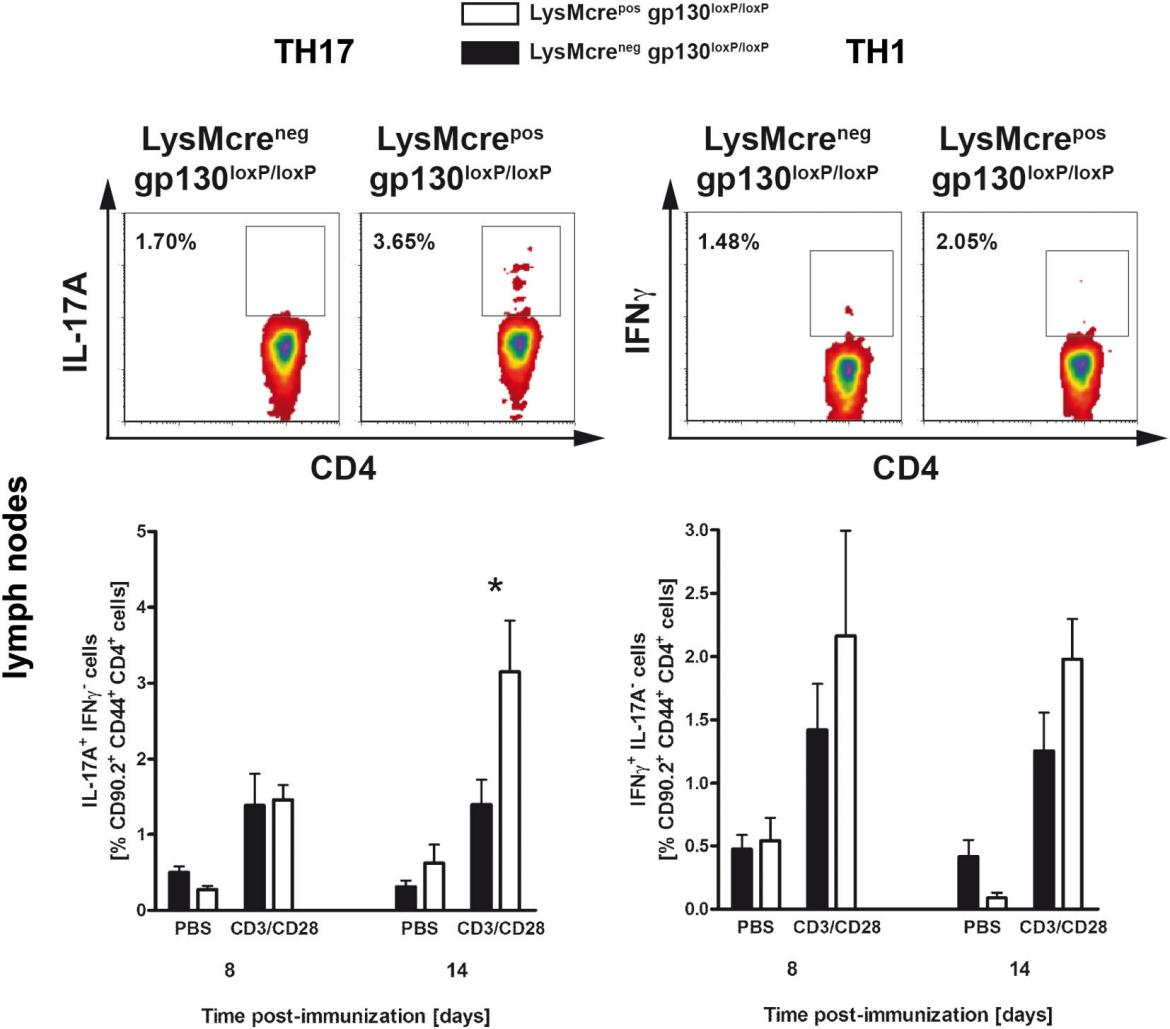
TH17 and TH1 immune responses in immunized LysMcre^pos^gp130^loxP/loxP^ mice and LysMcre^neg^gp130^loxP/loxP^ littermates. During the course of EAE, the frequency of TH17 and TH1 cells was determined by intracellular cytokine staining and subsequent flow cytometry. Analysis of intracellular IL-17A (left) and IFNγ production (right) by anti-CD3/CD28-stimulated cells is shown. For the analysis of TH17 and TH1 cells, single cell suspensions of the draining lymph nodes were performed at different time points (gated on CD90.2^+^CD44^+^CD4^+^ cells). Representative flow cytometry density plots of IL-17A- and IFNγ-producing cells isolated on day 14 are shown. Data represent means and SEs of five mice (day 8) and at least four mice (day 14) per group, respectively. One experiment representative out of two performed is shown. Statistical analysis was performed using Mann–Whitney test. Asterisks indicate statistical significance (*p ≤ 0.05).

To analyze the impact of macrophage/neutrophils-gp130 on the initial development of pathogenic T cells during the course of EAE, the frequency of TH1 and TH17 cells in lymphoid organs was determined by flow cytometry analyses (Figure 5). Whereas 8 days after immunization, the frequency of TH17 cells was still comparable in both groups, 14 days after immunization LysMcre^pos^gp130^loxP/loxP^ mice show significantly more TH17 cells compared with LysMcre^neg^gp130^loxP/loxP^ littermates (3.1 versus 1.4%). Hence, only in LysMcre^pos^gp130^loxP/loxP^ mice the frequency of TH17 cells increased during the course of EAE whereas the frequency of TH17 cells in immunized LysMcre^neg^gp130^loxP/loxP^ mice remained unchanged. The development of TH1 cells was not significantly different in immunized LysMcre^pos^gp130^loxP/loxP^ mice compared with LysMcre^neg^gp130^loxP/loxP^ littermates. Therefore, these results illustrate that the deletion of gp130 on macrophages/neutrophils amplified the development of inflammatory TH17 cells after EAE induction as early as 14 days after immunization.

### Immunized LysMcre^pos^gp130^loxP/loxP^ Mice Show Enhanced CNS-Infiltration

**Figure 6:**
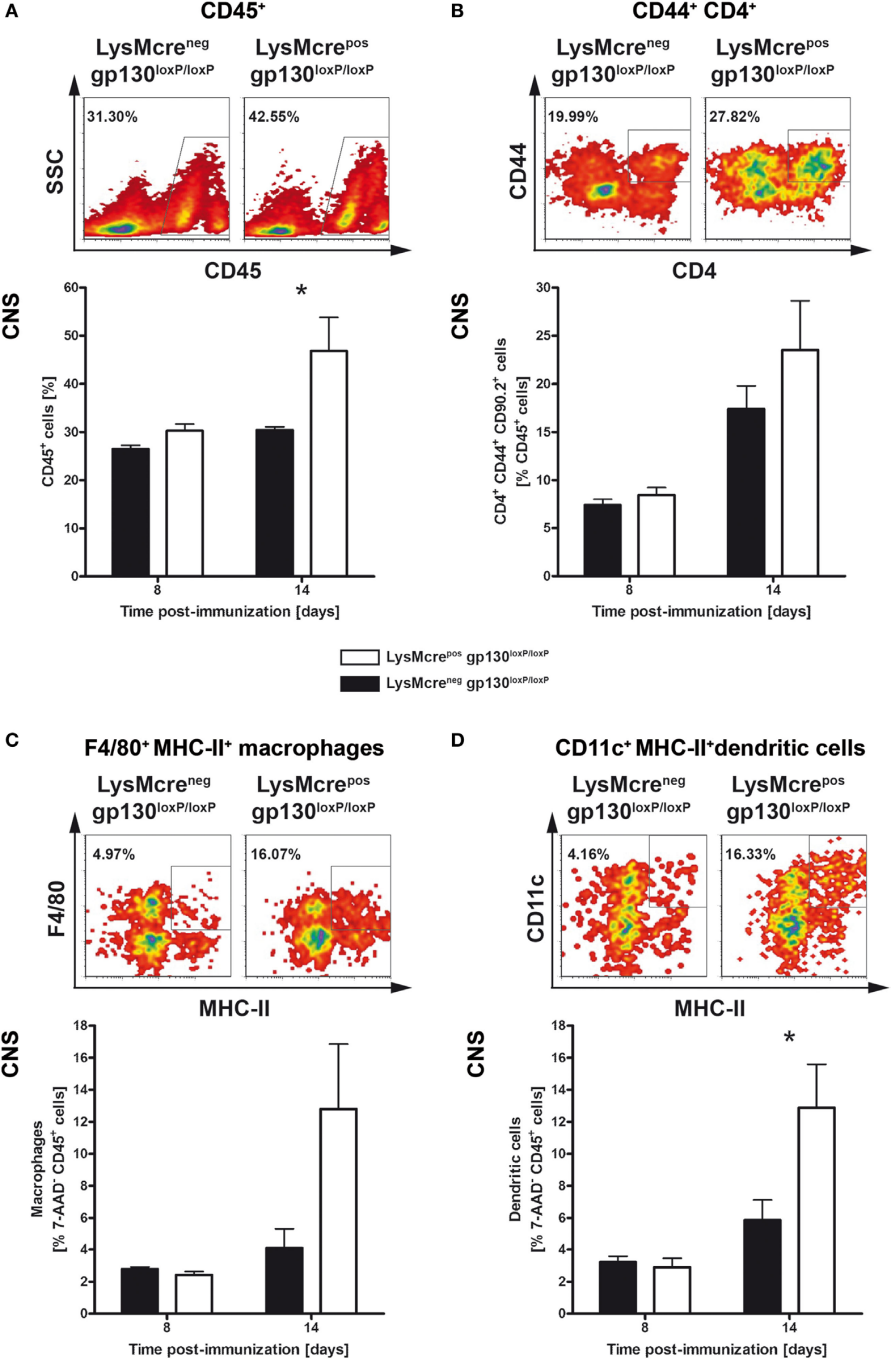
Characterization of infiltrates in immunized LysMcre^pos^gp130^loxP/loxP^ mice and LysMcre^neg^gp130^loxP/loxP^ mice. LysMcre^pos^gp130^loxP/loxP^ mice (white bars) and LysMcre^neg^gp130^loxP/loxP^ littermates (black bars) were immunized with MOG_35–55_/CFA. During the course of EAE, the frequency of **(A)** CD45^+^ lymphocytes, **(B)** activated CD44^+^CD4^+^ T cells, **(C)** F4/80^+^MHC-II^+^ macrophages, and **(D)** CD11c^+^MHC-II^+^ dendritic cells was determined by flow cytometry. For the analysis, single cell suspensions of CNS-infiltrating lymphocytes were performed at different time points. Representative flow cytometry density plots of cells isolated on day 14 are shown. Data represent means and SEs of five mice per group. Statistical analysis was performed using Mann–Whitney test. Asterisks indicate statistical significance (*p ≤ 0.05).

After immunization with MOG_35–55_/CFA, activated leukocytes gain entry into the CNS *via* the blood–brain barrier (39–42). To investigate the effect of an initially increased frequency of inflammatory T cells in the periphery on CNS-infiltration, single cell suspensions of CNS-infiltrated cells were analyzed by flow cytometry analyses (Figure 6). The influx of CD45^+^ leukocytes (Figure 6A), CD4^+^ T cells (Figure 6B), F4/80^+^MHC-II^+^ macrophages (Figure 6C), and CD11c^+^MHC-II^+^ dendritic cells (Figure 6D) was examined 8 and 14 days after EAE induction, respectively. Fourteen days after immunization, the frequency of CD45^+^ leukocytes was significantly increased in immunized LysMcre^pos^gp130^loxP/loxP^ mice compared with cre-negative littermates (Figure 6). The frequency of CD4^+^CD44^+^CD90.2^+^ T cells was not statistically different in these mice as well. Whereas the proportion of F4/80^+^MHC-II^+^ macrophages was also not statistically increased, the frequency of CD11c^+^MHC-II^+^ dendritic cells in all viable CD45^+^ cells was clearly enhanced in immunized LysMcre^pos^gp130^loxP/loxP^ mice. Together, the enhanced inflammatory immune response in peripheral organs of immunized LysMcre^pos^gp130^loxP/loxP^ mice led to an increased infiltration of CD45^+^ leukocytes and CD11c^+^MHC-II^+^ dendritic cells into the CNS already 14 days after immunization.

### Immunized LysMcre^pos^gp130^loxP/loxP^ Mice Show Significantly Increased Frequency of TH17 and TH1 Cells Within the CNS

**Figure 7:**
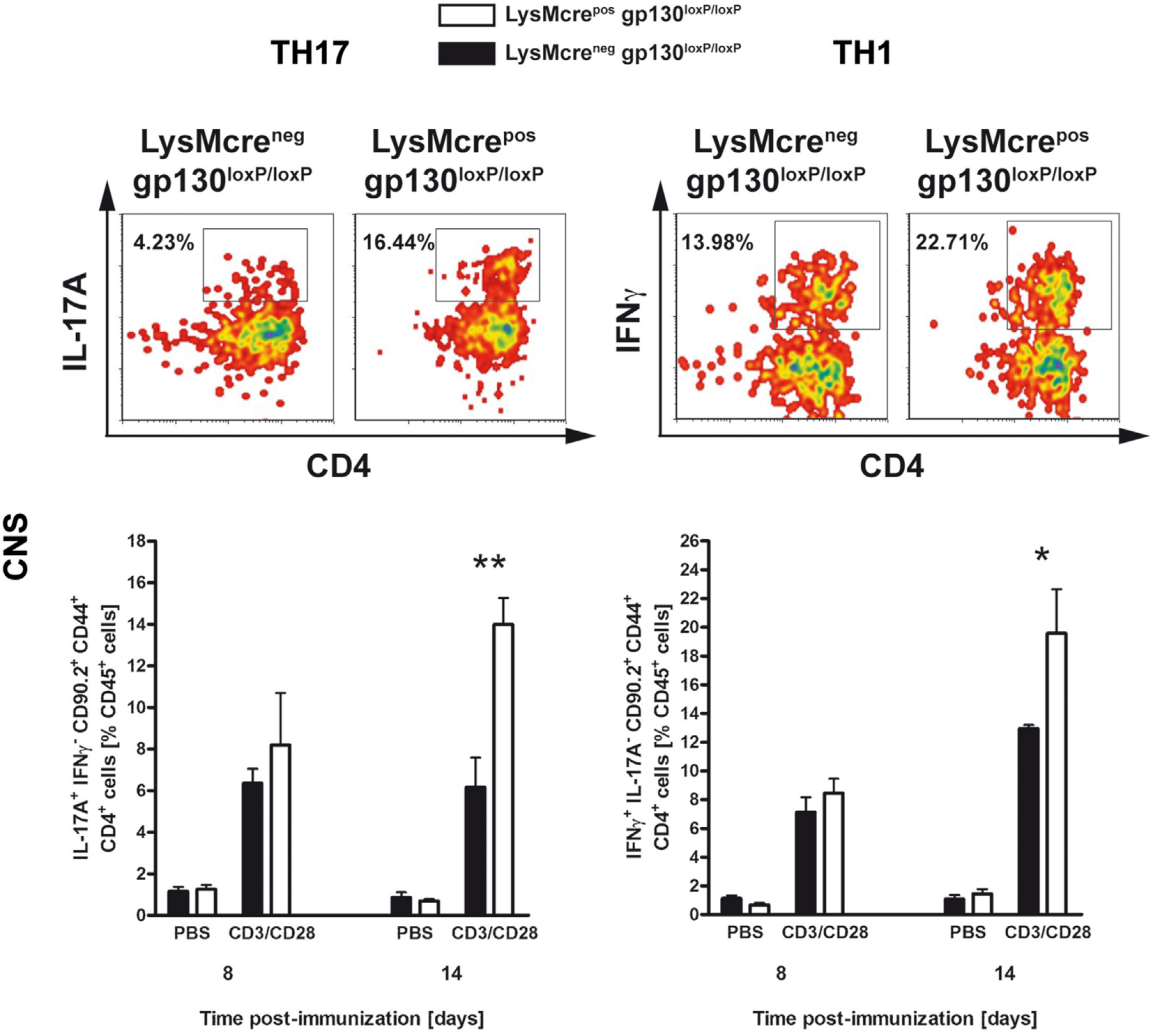
TH17 and TH1 immune responses in the CNS of immunized LysMcre^pos^gp130^loxP/loxP^ mice and LysMcre^neg^gp130^loxP/loxP^ littermates. During the course of EAE, the frequency of CNS-infiltrating TH17 and TH1 cells was determined by intracellular cytokine staining and subsequent flow cytometry. Analysis of intracellular IL-17A (left) and IFNγ production (right) by anti-CD3/CD28-stimulated cells is shown. For the analysis of TH17 and TH1 cells, single cell suspensions of CNS-infiltrating lymphocytes were performed at different time points (gated on CD90.2^+^CD44^+^CD4^+^ cells). Representative flow cytometry density plots of IL-17A- and IFNγ-producing cells isolated on day 14 are shown. Data represent means and SEs of five mice per group. One experiment representative out of three performed is shown. Statistical analysis was performed using Mann–Whitney test. Asterisks indicate statistical significance (*p ≤ 0.05; **p ≤ 0.01).

To analyze early CNS-infiltrated CD4^+^CD44^+^CD90.2^+^ T cells in more detail, intracellular staining of IL-17A and IFNγ in CNS-isolated lymphocytes was performed. At time of disease onset, 14 days after immunization, immunized LysMcre^pos^gp130^loxP/loxP^ mice show significantly increased frequency of infiltrated TH17 and TH1 cells compared with cre-negative littermates (Figure 7). In summary, this pathological infiltration reflected the subsequent clinical characteristics of immunized LysMcre^pos^gp130^loxP/loxP^ mice. Moreover, these results demonstrated that the deletion of macrophage/neutrophil-gp130 has an impact on the induction and progression of EAE.

### Expression of TH17-Driving Cytokines and Pro-Inflammatory Mediators Within the CNS of Immunized LysMcre^pos^gp130^loxP/loxP^ Mice

**Figure 8:**
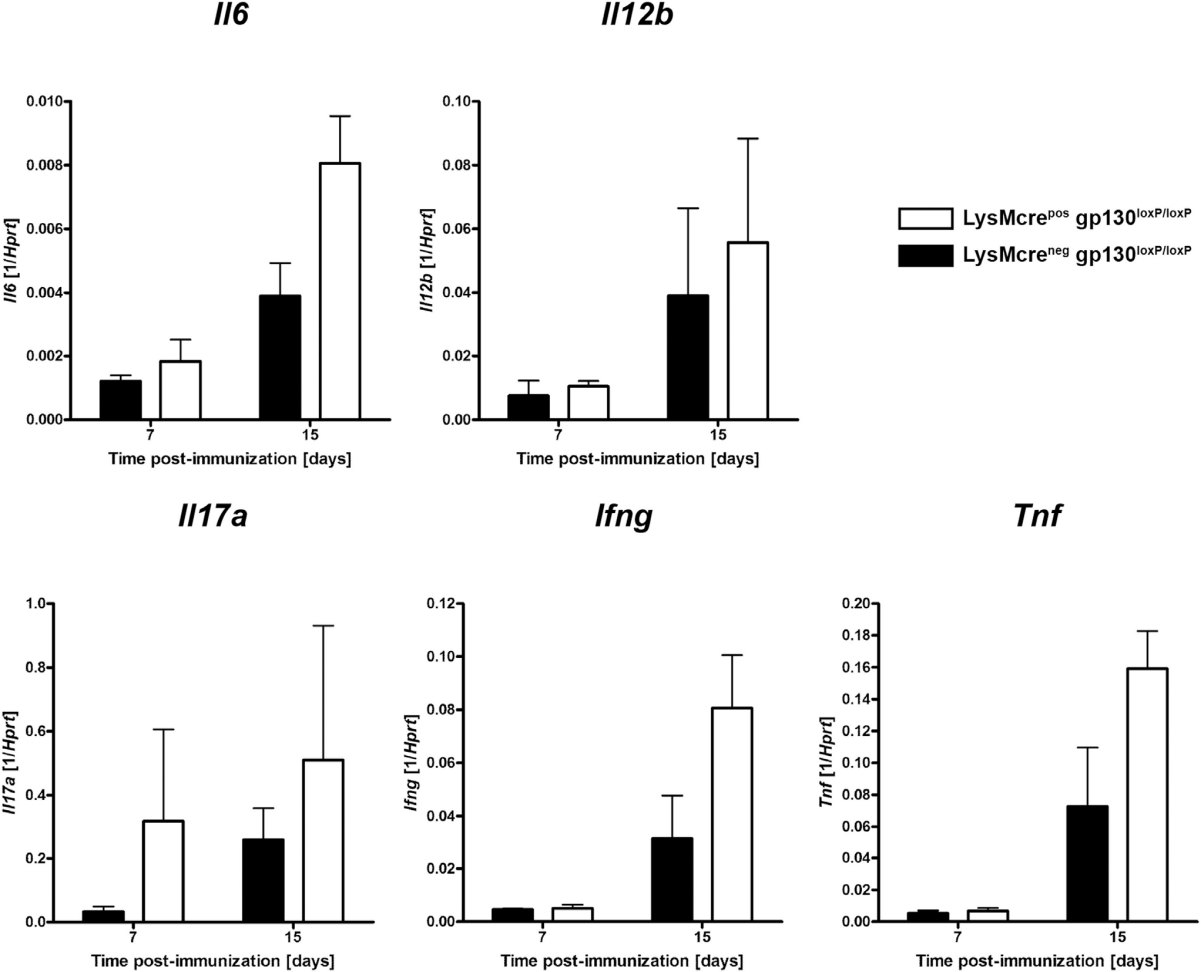
Expression of cytokines during the course of EAE. Immunized LysMcre^pos^gp130^loxP/loxP^ mice (white bars) show enhanced expression of *Il6, Il12b, Ifng, Il17a*, and *Tnf* compared with LysMcre^neg^gp130^loxP/loxP^ littermates (black bars). At different time points after EAE induction, CNS homogenates were assayed for gene expression by quantitative real-time RT-PCR. Data are expressed as ratio of induced factors versus endogenous *Hprt* and expressed as means and SEs of three mice per group. Statistical analysis was performed using Mann–Whitney test.

To assess the immune response within the CNS of immunized LysMcre^pos^gp130^loxP/loxP^ and LysMcre^neg^gp130^loxP/loxP^ mice at the onset of disease, gene expression studies were performed. At different time points early after EAE induction, CNS homogenates were assayed for gene expression of *Il6, Il12b, Ifng, Il17a*, and *Tnf* by quantitative real-time RT-PCR (Figure 8). Fifteen days after immunization, LysMcre^pos^gp130^loxP/loxP^ mice showed no statistically higher expression of the TH17-driving cytokine IL-6 as LysMcre^neg^gp130^loxP/loxP^ littermates. The *Il12b*-expression was comparable in both groups.

Furthermore, as marker for an inflammatory immune reaction, we also monitored the expression of *Il17a, Ifng*, and *Tnf*. Seven as well as 15 days after immunization LysMcre^pos^gp130^loxP/loxP^ mice showed no significantly enhanced expression of IL-17A compared with LysMcre^neg^gp130^loxP/loxP^ littermates. In addition, 15 days after immunization gene expression of the pro-inflammatory cytokines, *Ifng* and *Tnf* were also not significantly increased, respectively. Together, early after immunization, the inflammatory immune response in the CNS was not significantly elevated in LysMcre^pos^gp130^loxP/loxP^ mice.

## Discussion

gp130 cytokines, which are mainly expressed by antigen-presenting cells upon activation, differentially modulate the development of inflammatory immune responses *in vivo*. Within the gp130 cytokine family the monomeric cytokine IL-6 as well as the heterodimeric cytokine IL-27, composed of p28 and EBI-3, represent the most relevant members during T cell activation. Based on *in vitro* studies, pro-inflammatory properties of IL-6 as well as anti-inflammatory effects of IL-27 during TH17 cell differentiation are attributed to a differential signal transduction in naive T cells. Recently, it has been shown that the ability to access signal transducers and activators of transcription (STAT) 1 is responsible for the opposing effects of IL-6 and IL-27 on T cells (43). The importance of gp130 cytokines in EAE pathogenesis has persuasively been shown utilizing mice with systemic deletions of IL-6, IL-27Rα, and EBI-3, respectively (9–11, 19, 24, 44). In addition to T cells, activated macrophages also express gp130 together with the cytokine-specific receptor subunit IL-6R and IL–27Rα (26–29, 31, 32). However, the autocrine effect of gp130 signal transduction in activated macrophages is poorly investigated, although these cells play key roles in controlling the development of T cells through antigen presentation, expression of co-stimulatory receptors, and *via* the secretion of inflammatory mediators. Thus, the analysis of cell type-specific effects mediated by gp130 cytokines is essential to prevent negative side effects of immunotherapy targeting IL-6- or IL-27-signaling pathways.

In this study, immunized T cell-specific gp130-deficient mice were devoid of CNS-infiltration due to reduced frequencies of TH17 and TH1 cells in peripheral organs. Therefore, on the level of T cells, gp130 expression mediates CNS-infiltration and causatively induces EAE by particularly promoting the early development of pathogenic TH17 cells, and the outcome of EAE in T cell-specific gp130-deficient mice reflects the course of EAE in systemic IL-6-deficient mice (9–11). IL-6 could either directly promote the development of autoreactive TH17 cells (45–47) or indirectly by suppressing the effect of T_reg_ (12, 48). Hence, in our study, the TH17-polarizing effect of IL-6 could be attributed to both, the direct effect of IL-6 through T cell-gp130 and to the suppressive effect of IL-6 on T_reg_.

As gp130 cytokines, IL-6 and IL-27, have divergent effects on TH17 differentiation. In contrast to IL-6, IL-27 directly inhibits the development and effector response of TH17 cells (19, 21, 49). With respect to this putative T cell-gp130-mediated anti-inflammatory effect through IL-27, however, we cannot conclude from our data that IL-27 directly suppresses TH17 cells in EAE because the absence of IL-6-mediated signaling in CD4cre^pos^gp130^loxP/loxP^ mice impedes TH17 development. In summary, using T cell-specific gp130-deficient mice our data point to the T cell-specific impact of IL-6 through gp130 on the development of autoreactive TH17 cells and the pathogenesis of EAE.

In addition to T cells, activated macrophages and neutrophils likewise express functional receptor complexes for IL-6 as well as IL-27. Therefore, IL-6 and IL-27 indirectly influence T cell immune responses by regulating the effector function of activated macrophages and neutrophils. In contrast to T cells, it has convincingly been shown that IL-6- and IL-27-dependent signal transduction in macrophages lead to inhibition and to reduced secretion of inflammatory cytokines (26–29, 31, 32). Moreover, upon activation, myeloid cells play a crucial role in the development of tissue inflammation and autoimmunity (50–52). Initial experiments suggest that macrophage-gp130 modulates TH17 cell development by downregulating the secretion of inflammatory mediators (31). A recent study revealed that the development of autoreactive T cells and subsequent EAE pathogenesis is controlled by the IL-27-mediated suppression of dendritic cell functions (53). Using the nephrotoxic nephritis model of acute crescentic glomerulonephritis recent studies could show that IL-6-mediated dampening of macrophage activation protects tissues from overshooting immune responses (38). The gp130 cytokines IL-6 and IL-27 may also modulate inflammation through gp130 on neutrophils. The effect of IL-6 on neutrophils, however, appears to be pro- and anti-inflammatory (54–56). By contrast, IL-27 has been described to clearly suppress neutrophil development and function (33–35). Cre-recombinase activity controlled by the lysozyme M promoter leads to deletion of loxP-flanked gene sequences in both macrophages and neutrophils (57). Because inflammatory macrophages (58–60) as well as inflammatory neutrophils (61–63) promote disease progression in EAE, both cell types may, when uncontrolled, contribute to the increased pathology in LysMcre^pos^gp130^loxP/loxP^ mice shown in this study. However, since distinct subpopulations of macrophages and neutrophils (i.e., alternatively activated macrophages and myeloid-derived suppressor cells) (64–66) rather act anti-inflammatory or suppressive during EAE, the lack of macrophage/neutrophil-gp130 may also hinder the activation and development of these protective cells.

Our present study suggests that gp130 cytokines suppress EAE pathogenesis on the level of macrophages and/or neutrophils. A closer examination of the kinetic effects in the periphery and the CNS of immunized LysMcre^pos^gp130^loxP/loxP^ mice indicates that the macrophage/neutrophils-gp130-mediated effect on TH17 development in the draining lymph node and the leukocyte infiltration in the CNS proceeds at a rather late time point when first EAE symptoms already appear. This delayed increase in inflammation indicates that gp130 on macrophages/neutrophils does not suppress the initial development of TH17 cells and inflammation. It rather limits the accumulation of TH17 cells and leukocytes in the periphery and the CNS at the onset of the chronic disease, respectively. At this time point, the inflammation in the CNS and the disease was not significantly different in LysMcre^pos^gp130^loxP/loxP^ mice and cre-negative littermates. However, the disease began to worsen and secondary to the increased accumulation of TH17 cells, and leukocytes symptoms were significantly elevated in the absence of macrophage/neutrophil-gp130 when the clinical score was decreasing in gp130-competent mice. Hence, gp130 signaling in macrophages/neutrophils appears to maintain CNS inflammation indirectly through the eventual accrual of TH17 cells and leukocytes and/or directly by modulating the effector response in macrophages/neutrophils.

Nonetheless, because macrophage/neutrophil-specific gp130-deficient mice develop an aggravated course of EAE compared with control littermates but not macrophage/neutrophil-specific IL-6R-deficient mice this regulatory effect on macrophages is mediated by gp130 cytokines independently of IL-6. In line with our observation, IL-6 has recently been shown to promote the development of pathogenic TH17 cells in EAE by IL-6-trans-presentation to T cells through the IL-6R on dendritic cells (67). Overall, gp130 cytokines control pathogenic immune responses not solely *via* T cell-gp130 but also by regulating activated macrophages/neutrophils.

Recent studies revealed that CNS-dependent gp130 expression on astrocytes and neurons support cell survival and contribute to EAE pathogenesis (68–70) and therefore give evidence that cell type-specific effects of gp130 cytokines differentially contribute to the regulation of autoreactive inflammatory processes. However, additional experiments are needed to clarify which cells in the CNS are responsive to gp130 cytokines. In this context, our experiments demonstrate that gp130 cytokines possess cell type-specific effects on T cells and macrophages/neutrophils. Whereas for example IL-6 preferentially leads to the phosphorylation of STAT3 dimers, IL-27 mainly induces the formation of phosphorylated STAT1-monomers and STAT1/3-dimers (49, 71). On a functional level, the differential consequences of T cell-gp130 and macrophage/neutrophil-gp130 signaling during EAE can therefore be explained by distinct phosphorylation of intracellular STAT proteins and thus the induction of different transcription factors. In addition, a cell type-specific ability to access STAT1 may also be responsible for the differential effect of gp130-mediated signaling in CD4 T cells and macrophages (43).

In addition to IL-6 and IL-27, other cytokines also belong to the gp130 cytokine family including IL-35, IL-11, leukemia inhibitory factor (LIF), oncostatin m, ciliary neurotrophic factor, and cardiotrophin-1 (CT-1) (72–74). Also the IL-27p28 subunit (IL-30) induces STAT3-mediated signals independently of the IL-27Rα through the IL-6R *via* a gp130 dimer (75). However, not much is known about the pro-inflammatory or anti-inflammatory effect on myeloid cells and on the level of T cells. Solely IL-6 and IL-27 represent the most relevant members in modulating the contrarian development of TH17 cells and T_reg_. Only LIF and IL-35 have also been shown to inhibit TH17 immune responses. This anti-inflammatory property of the gp130 cytokine LIF favors T_reg_ commitment (76), controls TH17 cells and—produced by neural progenitor cells—improves EAE (77). Nevertheless, a macrophage/neutrophil-specific anti-inflammatory function in this respect has not been described for LIF. In addition, recent studies also suggest IL-35 which is mainly produced by T_reg_ but also by B cells (78, 79) as a gp130 cytokine that has a striking regulatory impact on TH17 cells. However, the exact role of IL-35 in the context of gp130-modulated CNS inflammation *in vivo* is still unclear (44, 79). Considering that IL-27 is produced by myeloid cells whereas the lymphoid compartment secretes IL-35, adoptive transfer experiments suggest that rather IL-27 but not IL-35 is capable to suppress the development of EAE (44). Moreover, delivery of IL-27 but not of IL-35 through a gene therapeutic approach ameliorates neuropathology in EAE (80). Hence, great evidence exists that IL-27 has at least a superior anti-inflammatory function in EAE over IL-35. Several studies indicate that IL-27 suppress the release of TH17- and TH1-driving cytokines by myeloid-derived cells (27–29, 31). In this respect, the course of EAE in macrophage/neutrophil-specific gp130-deficient mice reflects the elevated progression of EAE in immunized IL-27Rα-deficient mice (24). Therefore, the present results obtained from macrophage/neutrophil-specific gp130-deficient mice also suggest that the increased development of autoreactive T cells in immunized IL-27Rα-deficient mice is at least in part mediated by an anti-inflammatory effect of IL-27 on macrophages/neutrophils and may not be limited to the suppressive function of IL-27 on TH17 cells.

In summary, gp130 on T cells promotes TH17 development and accounts subsequently for pathogenesis in EAE. Notably, our data demonstrate that gp130 cytokines ameliorate disease progression during the course of EAE on the level of macrophages/neutrophils and second illustrate that gp130 cytokines exert cell type-specific effects which influence T cell development substantially. Hence, in regard to improve effectiveness of immunomodulatory therapeutics, which aims to treat TH17-mediated diseases such as MS the analysis of cell type-specific effects is essential. Otherwise, systemic targeting of gp130-mediated signaling pathways to suppress TH17 cell differentiation may incur the risk of an unwanted release of pro-inflammatory cytokines by activated macrophages/neutrophils, which presumably promote an inflammatory milieu.

## Materials and Methods

### Mice

Breeding pairs of CD4cre^pos^gp130^loxP/loxP^ and LysMcre^pos^gp130^loxP/loxP^ mice (57, 81) were kindly provided by Werner Müller (Braunschweig, Germany) and bred under specific pathogen-free conditions at the Research Center Borstel. IL-6R^loxP/loxP^ mice were generated as previously described (82), and LysMcre^pos^IL-6R^loxP/loxP^ mice were raised at the animal facility of the University Medical Center Hamburg-Eppendorf. Experimental mice were between 8 and 12 weeks old. In any given experiment, mice were matched for age, sex, and genetic background. All experiments performed were in accordance with German Animal Protection Law and were approved by the Animal Research Ethics Board of the Ministry of the Environment, Kiel, Germany.

### Induction and Assessment of EAE

Mice were injected subcutaneously with 100 µg of MOG_35–55_ peptide (MEVGWYRSPFSRVVHLYRNGK) emulsified in CFA supplemented with 2 mg/ml killed *Mycobacterium tuberculosis* H37Ra and injected twice intravenously with 300 ng of pertussis toxin (both Hooke Laboratories). Clinical assessment of EAE was performed daily after disease induction according to the following criteria: 0, no disease; 1, decreased tail tone; 2, hindlimb weakness or partial paralysis; 3, complete hindlimb paralysis; 3, 5, forelimb and hindlimb paralysis; 4, moribund state.

### Preparation of Single Cell Suspensions

Single cell suspensions of CNS-infiltrates, lymph nodes, and spleen were prepared at different time points. Mice were sacrificed with CO_2_ and subsequently perfused with cold PBS. Brain tissue and spinal cord were treated with collagenase A (1 mg/ml, Roche) and DNase I (30 µg/ml, Sigma) for 30 min at 37°C, disrupted by passage through a nylon cell strainer and subsequently isolated by density gradient centrifugation (Percoll, GE Healthcare). Lymph nodes and spleens were strained through 70 µm pore size nylon cell strainer. Recovered vital cells were counted using an automatic cell counter (ViCell^®^; Beckman Coulter), diluted in complete Iscove’s-modified Dulbecco’s medium (IMDM; Gibco) supplemented with 10% FCS (Gibco), 0.05 mM β-mercaptoethanol (Sigma), and penicillin and streptomycin (100 U/ml and 100 µg/ml; Gibco) and used for further experiments.

### Flow Cytometric Analyses

Cells were stained with primary antibodies to CD45 (BD Biosciences), CD4 (BD Biosciences), CD90.2 (BioLegend), CD44 (BioLegend), F4/80 (BioLegend), MHC-II (eBioscience), and CD11c (BioLegend) for 45 min at 4°C. Non-specific antibody binding was blocked by incubation with a cocktail containing anti-FcγRIII/II mAb (clone 2.4G2), mouse, and rat sera.

### Intracellular Cytokine Staining

For detection of intracellular IFNγ and IL17A cells were incubated with IMDM or stimulated with plate-bound anti-CD3/CD28 mAb (clone 2C11 and clone 37/51 at 10 µg/ml, respectively) for 4 h in the presence of GolgiPlug™ (BD Biosciences). After surface staining with antibodies to CD4, CD90.2, and CD44, cells were fixed and permeabilized with Cytofix/Cytoperm™ (BD Biosciences), and intracellularly accumulated IFNγ and IL-17A were stained. Activated lymphocytes were gated by CD4^+^CD90.2^+^ and CD44^+^ profile. Viable cells were identified by 7-AAD staining. Fluorescence intensity was analyzed using a FACSCanto II (BD Biosciences). Data were acquired with FACSdiva software (BD Biosciences).

### Histology

Histology was performed as described recently (83). Spinal cords were removed and fixed in 4% formalin-PBS. Subsequently, spinal cords were dissected, embedded in paraffin and sectioned (2–3 µm) before staining with Luxol fast blue (LFB-PAS) to assess the degree of demyelination. For the immunohistochemical detection of macrophages/microglia, T cells, B cells, and axonal damage tissue sections were deparaffinized and pressure cooked in 10 mM citrate buffer, pH 6.0. After peroxidase quenching with 3% H_2_O_2_/PBS and blocking with 10% FCS, sections were incubated with primary antibodies against MAC-3 (1:200, BD Bioscience) for macrophages and microglia, CD3 (1:100, MCA1477 Serotec, Düsseldorf, Germany) for T cells, B220 (1:200, BD Bioscience) for B cells, and APP (1:3,000, MAB348, Millipore) for indication of axonal damage overnight followed by the incubation with HRP-conjugated secondary antibodies. Development was performed by using DAB-buffer tablets (Merck, Darmstadt, Germany). Spinal cord sections were evaluated using cell-P software (Olympus).

### Quantitative Real-Time RT-PCR

Total RNA was extracted by acid phenol extraction. cDNA was obtained using murine moloney leukemia virus reverse transcriptase (Superscript II, Invitrogen) and oligo-dT (12–18mer; Sigma) as a primer. The following primer sets were used: *Hprt*: sense 5′-TCC TCC TCA GAC CGC TTT T-3′, antisense 5′-CCT GGT TCA TCA TCG CTA ATC-3′, probe 5′-AGT CCA G-3′, *Il6*: sense 5′-GTC ACC AAA CTG GAT ATA ATC AGG A-3′, antisense 5′-CCA GGT AGC TAT GGT ACT CCA GAA-3′, probe 5′-TTC CTC G-3′; *Il12b*: sense 5′-ATC GTT TTG CTG GTG TCT CC-3′, antisense 5′-GGA GTC CAG TCC ACC TCT ACA-3′, probe 5′-AGC TGG AG-3′; *Ifng*: sense 5′-ATC TGG AGG AAC TGG CAA AA-3′, antisense 5′-TTC AAG ACT TCA AAG AGT CTG AGG TA-3′, probe 5′-CAG-AGC CA-3′; *Il17a*: sense 5′-TGT GAA GGT CAA CCT CAA AGT CT-3′, antisense 5′-GAG GGA TAT CTA TCA GGG TCT TCA T-3′, probe 5′-GCT CCA GA-3′; *Gp91phox*: sense 5′-TGC CAC CAG TCT GAA ACT CA-3′, antisense 5′-GCA TCT GGG TCT CCA GCA-3′, probe 5′-ACC TGC TG-3′; *Tnf*: sense 5′-TCT TCT CAT TCC TGC TTG TGG-3′, antisense 5′-GGT CTG GGC CAT AGA ACT GA-3′, probe 5′-TGG TGG CC-3′. Quantitative PCR was performed on a Light Cycler (Roche Diagnostics Corporation). Data were analyzed employing the “Fit Points” and “Standard Curve Method” using *hypoxanthine-guanine phosphoribosyle transferase* (*Hprt*) as housekeeping gene to calculate the level of gene expression in relation to *Hprt*.

### Statistical Analysis

Quantifiable data are expressed as mean and SE. After analyzing for Gaussian distribution the Mann–Whitney test was applied, defining different error probabilities (*p ≤ 0.05, **p ≤ 0.01).

## Ethics Statement

All experiments performed were in accordance with German Animal Protection Law and were approved by the Animal Research Ethics Board of the Ministry of the Environment, Kiel, Germany.

## Author Contributions

KH performed the experiments, analysed the data and wrote the manuscript. MP wrote the manuscript. SB performed experiments and analysed data. AH performed experiments and analysed data. FD performed experiments. H-WM provided material. SR-J provided material. CH conceived and designed the experiments and wrote the manuscript.

## Conflict of Interest Statement

The authors declare that the research was conducted in the absence of any commercial or financial relationships that could be construed as a potential conflict of interest.
