# Supplementary figures and images for: Differing Outcome of Experimental Autoimmune Encephalitis in Macrophage/Neutrophil- and T Cell-Specific gp130-Deficient Mice
Source: Front Immunol. 2018 May 2;9:836. doi: 10.3389/fimmu.2018.00836 (PMC5940746; doi:10.3389/fimmu.2018.00836)

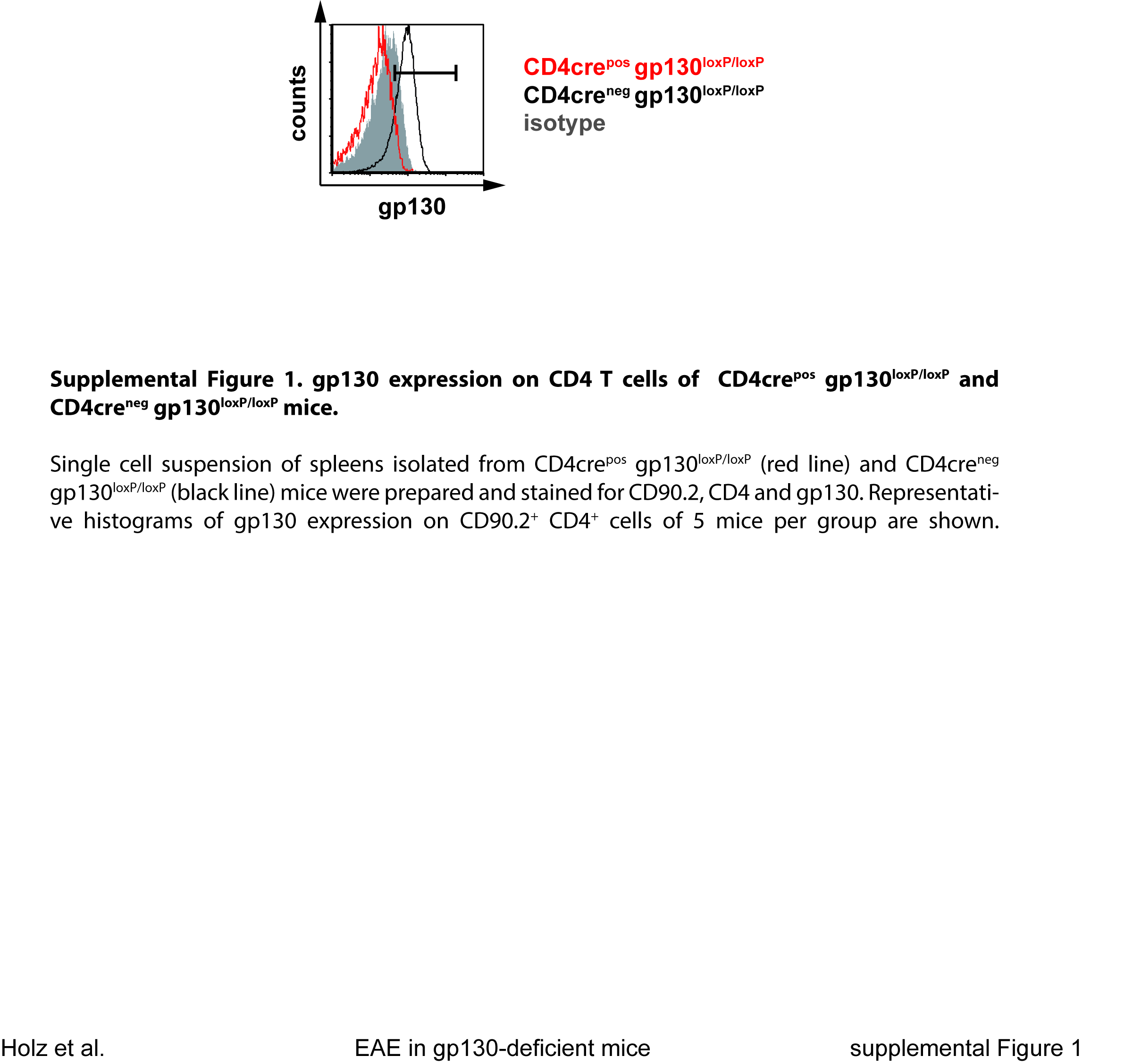

Supplement: Supplementary file 1 [file image_1.tif]
